# Characterization of serum follicle stimulating hormone, growth hormone, leptin, luteinizing hormone, and prolactin in female polar bears (*Ursus maritimus*) via multiplex immunoassay

**DOI:** 10.1093/jas/skag273

**Published:** 2026-08-31

**Authors:** Jessye Wojtusik, Emily E Virgin, Erin Curry

**Affiliations:** Department of Reproductive Sciences, Omaha’s Henry Doorly Zoo & Aquarium, Omaha, NE 68107, United States; Department of Reproductive Sciences, Omaha’s Henry Doorly Zoo & Aquarium, Omaha, NE 68107, United States; Cincinnati Zoo & Botanical Garden, Center for Conservation and Research of Endangered Wildlife, Cincinnati, OH 45220, United States

**Keywords:** conservation, hormone monitoring, protein hormone, serum, threatened species, wildlife

## Abstract

Reproductive failure is an increasing concern for polar bear (*Ursus maritimus*) populations. Although hormone monitoring has improved understanding of reproductive function, serum-based assessments have been underutilized. This study evaluated the use of multiplex immunoassays to characterize key hormones in females. Serum samples (*n* = 103; 2–8 per bear) were collected from 25 females and used to characterize follicle stimulating hormone (FSH), growth hormone (GH), leptin, luteinizing hormone (LH), and prolactin (PRL) using commercial panels. Assay validation confirmed biologically meaningful profiles parallelism, acceptable spike-and-recovery (range: 91.6–99.8%), and analyte stability across four freeze-thaw cycles (<15% difference). Age groups were defined as juvenile (0–5.0 y), young adult (5.1–12.0 y), adult (12.1–20.0 y), and senior (>20.0 y). Juveniles were excluded from analyses except where age group was evaluated. Seasons were classified based on northern hemisphere astronomical seasons. FSH was frequently below the assay’s minimum detectable limit (MDL), indicating that although assay specificity was confirmed, the sensitivity was insufficient to reliably quantify physiological concentrations in this species. Detectable values (range: 7.8 to 22.4 ng/mL) were mostly restricted to the breeding season, with no samples above the MDL from May through November. GH concentrations differed significantly by season (*P *= 0.001), with the greatest model-adjusted mean during winter (4.29 ± 2.0 ng/mL) and lowest during summer (0.59 ± 0.28 ng/mL). Leptin concentrations differed by age group (*P *= 0.001), with the greatest model-adjusted mean in seniors (1.69 ± 0.50 ng/mL) and lowest in young adults (0.58 ± 0.42 ng/mL). LH also varied by age (*P *= 0.0002), with seniors showing the greatest model-adjusted mean (0.65 ± 0.18 ng/mL) and immature bears, the lowest (0.16 ± 0.04 ng/mL). PRL model-adjusted mean peaked in winter (38.05 ± 9.71 ng/mL) and spring (36.38 ± 8.49 ng/mL; *P *= 0.0001) and age group (*P *= 0.04); however, post-hoc tests revealed no significant difference in pairwise comparisons among the age groups. These results demonstrate the applicability of multiplex technology for reproductive hormone monitoring in polar bears. Notably, this study provides the first comprehensive characterization of several key hormones in female polar bears.

## Graphical abstract

**Figure:**
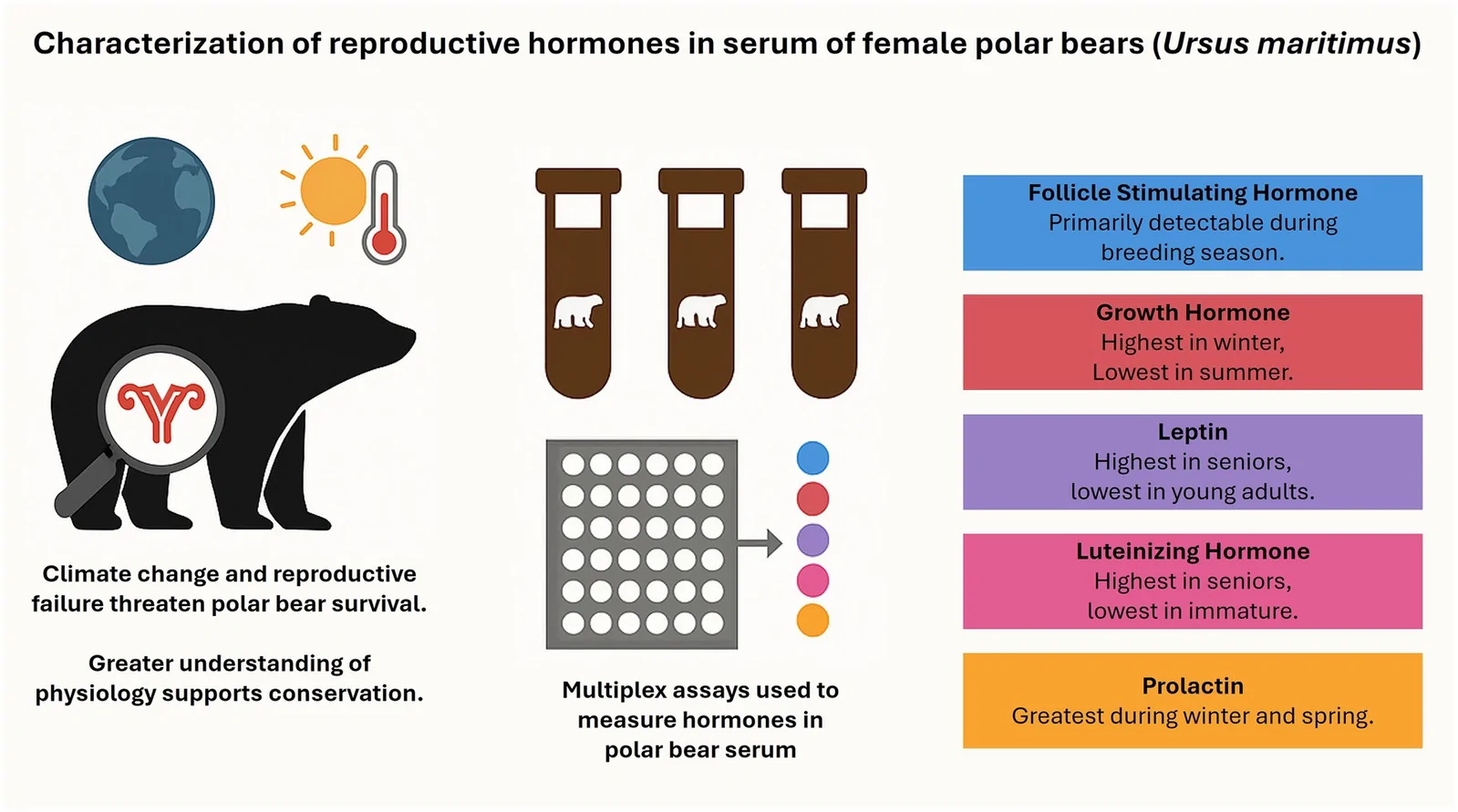

## Teaser

By validating multiplex assays for key reproductive hormones, this study provides a powerful new tool for monitoring reproductive physiology in female polar bears. The method has direct applications for zoo and conservation breeding programs.

## Introduction

Maintaining genetically sound and sustainable ex situ wildlife populations is a critical component of species conservation. These populations offer unique opportunities to advance physiological knowledge and develop tools that support animal health, longevity, and wellbeing (Pukazhenthi and Wildt 2004; Ryser-Degiorgis 2013; Braverman 2014). However, studying endangered or threatened wildlife presents unique challenges not commonly encountered in livestock or laboratory animal research. Regulatory restrictions can limit sample collection and transport, while small population sizes constrain sample diversity and availability (Ward et al. 2018; Shaw et al. 2021). In addition, practical barriers such as limited voluntary participation, low sample volumes, and restricted access to specialized tools can further complicate research efforts (Ryser-Degiorgis 2013; Shaw et al. 2021).

Noninvasive hormone monitoring has, nevertheless, greatly advanced our understanding of wildlife physiology, particularly reproductive biology, in species under human care (Schwarzenberger and Brown 2013; Kumar and Umapathy 2019; Ghosal et al. 2023). Longitudinal endocrine studies have proven instrumental in guiding breeding management, diagnosing and mitigating disease and stress, and informing conservation strategies (Pukazhenthi and Wildt 2004; Schwarzenberger and Brown 2013; Cizauskas et al. 2015; Kumar and Umapathy 2019; Ghosal et al. 2023). Fecal hormone analysis is especially valuable due to its non-invasive nature and ease of collection; however, it has limitations. Protein hormones are prone to degradation during digestion, and fecal hormone concentrations often reflect physiological events that occurred days earlier, reducing their effectiveness for real-time monitoring.

In contrast, serum provides a more immediate reflection of endocrine activity and is preferable for evaluating protein hormones. Historically, logistical constraints have limited the use of blood sampling in many wildlife species, but recent progress in operant conditioning has enabled voluntary blood collection from a growing number of taxa, including polar bears (*Ursus maritimus*). This advancement has created new opportunities to expand the scope and depth of physiological monitoring in species where research has traditionally relied on fecal sampling. Advancements in multiplex immunoassay technology further enhance these opportunities by enabling the simultaneous quantification of multiple hormones from a single, small-volume sample. This reduces the amount of serum required, minimizes assay time, and facilitates the detection of complex hormone interactions through parallel data collection. For rare and endangered species, where both sample volume and availability are limited, this approach offers a means to conduct more comprehensive endocrine profiling and advance our understanding of reproductive mechanisms.

Despite ongoing conservation efforts, in the U.S., polar bears face reproductive challenges that threaten their long-term viability. The U.S. ex situ population is declining and no longer self-sustaining (Faust and Jungheim 2025). Anecdotal reports suggest high rates of reproductive failure, including unsuccessful reproduction of unclear etiology (e.g. failure to conceive or maintain pregnancy) as well as agalactia (Meyerson et al. 2019). Polar bears are seasonal breeders, mating as photoperiods increase and giving birth when daylight hours are short, a pattern that differs from many other long-day breeders such as horses (*Equus caballus*) and mink (*Mustela vison*; Sundqvist et al. 1989; Aurich 2011). Polar bears also experience delayed implantation and an apparent obligatory pseudo-pregnancy regardless of embryo presence (Stoops et al. 2012). The mechanisms regulating diapause, corpus luteum reactivation, and implantation in this species remain poorly understood, and environmental factors such as photoperiod, temperature, and human interaction may further influence reproductive success.

While reproductive steroid hormone dynamics have been relatively well studied in polar bears, using both fecal and serum analysis, opportunities to assess a broader range of hormones have been constrained by the limited availability of banked serum, and the challenges of obtaining repeated samples across individuals and seasons (Stoops et al. 2012; Curry et al. 2012a, 2012b; Wojtusik et al. 2024; Derocher et al. 1992; Howell-Skalla et al. 2002; Tompros et al. 2025). As a result, many protein-based reproductive hormones remain uncharacterized in this species. Improved access to serum via voluntary sampling, together with the implementation of multiplex assays in polar bears (Bourque et al. 2020), provides a unique opportunity to assess a wider array of endocrine biomarkers and produce more comprehensive reproductive profiles.

This study had two primary objectives: (1) to validate the application of multiplex immunoassay technology for reproductive hormone analysis in a threatened wildlife species, and (2) to characterize serum biomarkers in polar bears, including follicle stimulating hormone (FSH), growth hormone (GH), leptin, luteinizing hormone (LH), and prolactin (PRL), selected based on their roles in reproductive physiology across mammals. We hypothesized that the multiplex assays would be validated and hormone concentrations would vary with age and season and reflect underlying reproductive status. These findings aim to inform breeding management strategies and support efforts to improve the sustainability of the U.S. ex situ polar bear population.

## Materials and methods

### Animals and sample collection

All procedures were reviewed and approved by the Cincinnati Zoo & Botanical Garden’s Animal Care and Use Committee (protocol #22-175 “Non-Invasive and Harmless Opportunistic Collection of Biological Samples from Animals”) and by participating institutions. Serum samples (*n* = 103) were collected opportunistically from 25 female polar bears (2 to 8 samples per female) during routine veterinary procedures or voluntary training sessions between 1986 and 2019. Samples were not collected specifically for the purposes of this study. Bears were housed at 15 institutions (1–2 bears per institution; latitude range: 32.7 to 45.5°, longitude range: −76.6 to −122.7°). Individuals ranged in age from 0 to 35 y at the time of sampling. All samples were stored at either −20 °C or –80 °C until analysis.

Samples (Supplementary File A) represented all calendar months and were selected to provide near even seasonal coverage, categorized according to Northern Hemisphere astronomical definitions: winter (December 21–March 21; *n* = 27), spring (March 22–June 21; *n* = 29), summer (June 22–September 24; *n* = 22), and fall (September 25–December 20; *n* = 25). Polar bear breeding season was defined as January 1–May 21 and non-breeding season as May 22–December 31, as described by Curry et al. (2012a, 2012b). Age classes (Tompros et al. 2025) were defined based on reproductive maturity and long-term health records as: immature (0–5.0 y; *n* = 24), young adult (5.1–12.0 y; *n* = 26), adult (12.1–20.0 y, *n* = 26), and senior (> 20.0 y; *n* = 27). Proven parity (yes; *n* = 43/no; *n* = 36) was assigned for mature individuals only, based on records indicating whether that individual had successfully produced offspring during or before a given year. Birth origin was classified as wild-born or captive-born.

### Multiplex assay and validation

Hormone concentrations were quantified using two magnetic bead-based multiplex immunoassay kits: the Milliplex Canine Pituitary Expanded Panel (CANPIT-96K; Millipore Sigma, Burlington, MA, USA) for measurement of adrenocorticotropic hormone (ACTH), FSH, GH, LH, PRL, and thyroid stimulating hormone (TSH); and the Milliplex Canine Gut Hormone Panel (CGTMAG-98K) for leptin. All samples were run neat (undiluted) in duplicate, and assays were performed according to the manufacturer’s protocols. Detection was conducted on a Luminex 200 platform, a bead-based flow cytometry multiplex immunoassay system.

To validate assay performance, a pooled serum sample was created by combining equal volumes from multiple individuals (*n* = 8) and was serially diluted to assess parallelism with standard curves of each analyte. An F-test was used to assess differences in the slopes of the binding curves. Accuracy was assessed by spiking the pooled sample with known concentrations of each hormone to calculate percentage recovery. Quality control samples were included on each plate and intra- and inter-assay coefficient of variation were maintained at <15%. Aliquots of the pooled sample were subjected to four freeze–thaw cycles (from −80 °C to 25 °C), with concentrations measured after each cycle to assess analyte stability. Samples with concentrations below the manufacturer’s minimum detectable level (MDL) were assigned the lowest detectable value for that analyte (Herbers et al. 2021; Tompros et al. 2025). MDL values were included in statistical analysis.

### Statistical analysis

All statistical analyses were performed using R (version 4.43, R Core Team). Hormone concentrations were natural log (ln)-transformed to meet assumptions of normality and homogeneity of variance. Normality and model assumptions were assessed using diagnostic plots (e.g. Q–Q plots and residual distributions) and were considered adequately met after transformation. For each hormone, two linear mixed-effects models were constructed using the “lme4” package (Bates et al. 2015): one including all females, and one limited to bears older than 5 y of age to reduce confounding by developmental immaturity. Fixed effects tested in each model included astronomical season (winter, spring, summer, fall), reproductive season (breeding or non), birth origin (wild vs. captive), age class (immature, young adult, adult or senior), and parity (proven or not). Individual bears were included as a random effect to account for repeated measures. Housing institution was excluded due to limited individuals per site (1–2 bears), with variation largely captured by individual random effects. Additionally, the relatively narrow geographic range represented (latitude: 32.7°–45.5°), and its alignment with institution, limited the ability to evaluate latitude-specific effects independently. Model selection was guided by Akaike’s Information Criterion, and non-significant variables (birth origin, reproductive season, and parity) were removed to improve model fit. Given overlap among calendar months and reproductive and astronomical seasons, astronomical season was used as the primary categorical variable.

Post-hoc comparisons were performed using “lmerTest” (Kuzenetsova et al. 2017), “modelbased” (Makowski et al. 2025) and “emmeans” (Lenth 2025). Graphs were created in R using “ggplot2” (Wickham 2016), “ggpubr” (Kassambara 2025), and “rstatix” (Kassambara 2023), and all plots display raw hormone values for interpretability. For each hormone, the raw data range and unadjusted mean are reported for descriptive context, whereas all statistical outcomes are derived from model estimates and presented as model-adjusted mean ± 95% confidence intervals. Statistical significance was set at α = 0.05.

## Results

Parallelism was confirmed between serially diluted pooled serum and the standard curves for each hormone analyte (FSH: F_2,2_ = 2.6; GH: F_2,2_ = 0.5; leptin: F_2,2_ = 1.5; LH: F_2,2_ = 1.4; PRL: F_2,2_ = 1.6, all F Test *P *> 0.05). Spike and recovery analysis yielded acceptable recovery rates for GH (99.8%), FSH (99.4%), leptin (91.6%), LH (96.2%), and PRL (98.9%). However, ACTH and TSH exhibited poor recovery (ACTH: 244.0%, TSH: 116.0%) and were excluded from further analysis. Freeze-thaw testing revealed no significant degradation for any analyte across four cycles (<15%; Figure S2). Birth origin and parity did not have a significant impact on any analyte.

### Follicle stimulating hormone (FSH)

In the majority of samples (98 out of 103) FSH fell below the assay’s MDL (0.95 ng/mL), precluding formal statistical analysis. Detectable raw values ranged from 7.8 to 22.4 ng/mL. Overall sample mean was 1.61 ± 0.32 ng/mL. Detectable values occurred only in winter (*n* = 4) and early spring (*n* = 1), with the greatest raw values observed in December (21.9 ng/mL) and January (16.0 ± 6.4 ng/mL). No samples from May through November exceeded the MDL, and models were not run for this hormone due to insufficient detectable data (Figure S1).

### Growth hormone (GH)

**Figure 1:**
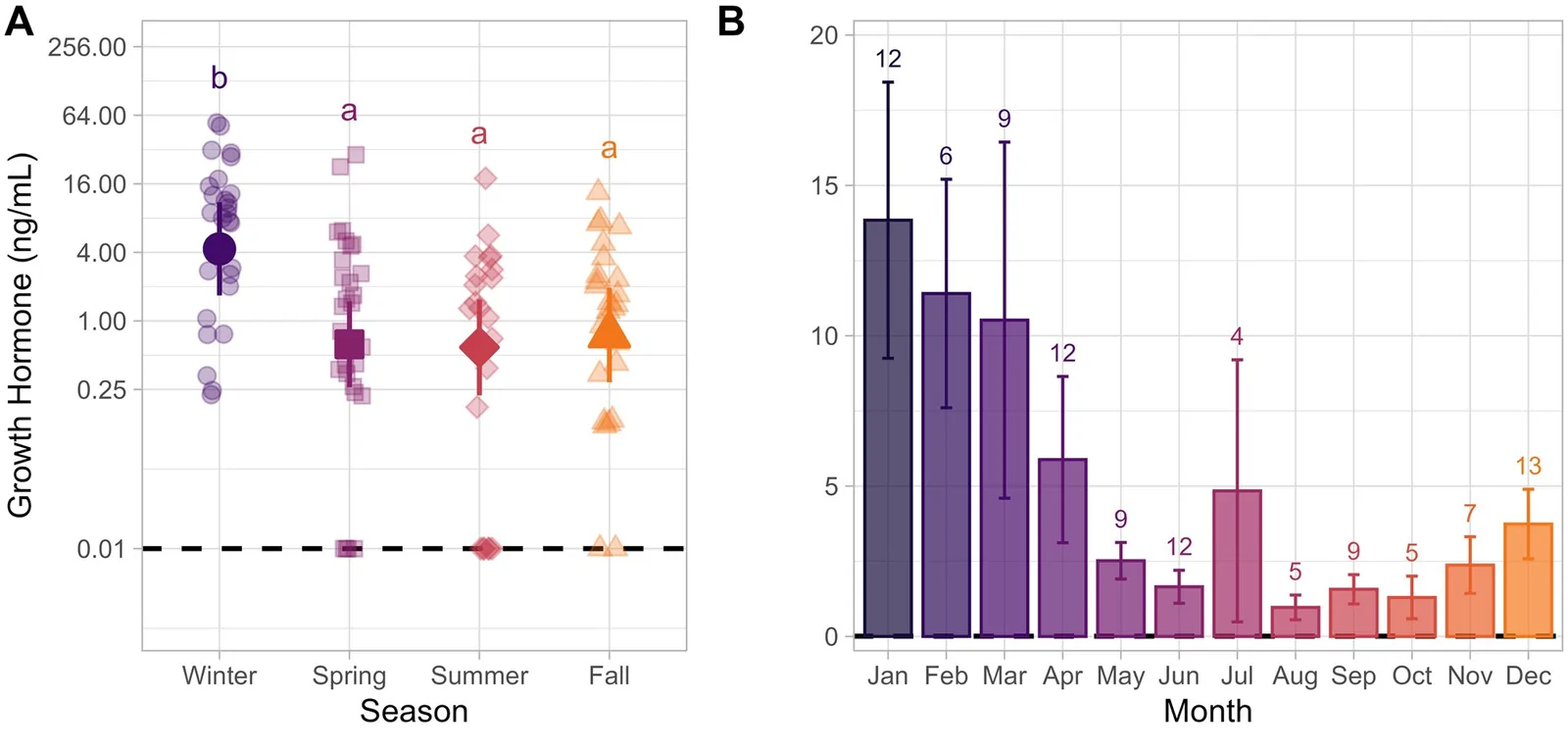
(A) Seasonal variation in growth hormone (GH) concentrations (ng/mL) in female polar bears (*Ursus maritimus*). Individual data points represent raw measurements across four seasons: Spring (circles), Summer (squares), Fall (diamonds), and Winter (triangles). The solid point in the middle of each error plot represents the model-adjusted means calculated from the corresponding linear mixed-effects model; the error bars represent the upper and lower 95% confidence limit. Different lowercase letters above error plots indicate statistically significant differences among groups (*P *< 0.05). Groups sharing the same letter are not significantly different (Winter vs. Spring, *P *= 0.004, Winter vs. Summer, *P *= 0.007; Winter vs. Fall, *P *= 0.04). (B) Monthly variation in GH concentrations (ng/mL) in female polar bears. Each bar represents mean monthly GH levels from January to December. Error bars represent ± standard error of measurement (SEM). Sample sizes for each month are indicated above the corresponding error bars. The dashed, horizontal line represents the minimum detectable limit (MDL; 0.01 ng/mL). No statistical analyses were performed for monthly comparisons.

GH concentrations were above the MDL (0.01 ng/mL) in nearly all samples, with only 12 samples at the MDL. Among the remaining samples, raw concentrations ranged from 0.13 to 54.91 ng/mL. The overall mean was 5.36 ± 0.94 ng/mL. GH concentrations were greatest in winter (model-adjusted means: 4.29 ± 2.02 ng/mL) and lower in spring (0.62 ± 0.27), summer (0.59 ± 0.28), and fall (0.75 ± 0.36; Figure 1A; overall model *P *= 0.001). The greatest GH values were observed in January through March, followed by a gradual decline throughout the remainder of the year (Figure 1B). GH levels were not impacted by age group (model-adjusted means: immature: 1.23 ± 0.63 ng/mL, young adult: 1.15 ± 0.55, adult: 0.73 ± 0.34, senior: 1.14 ± 0.57; overall model *P *= 0. 78) or parity (proven: 1.12 ± 0.56 ng/mL, not proven: 0.89 ± 0.49; overall model *P *= 0.83).

### Leptin

**Figure 2:**
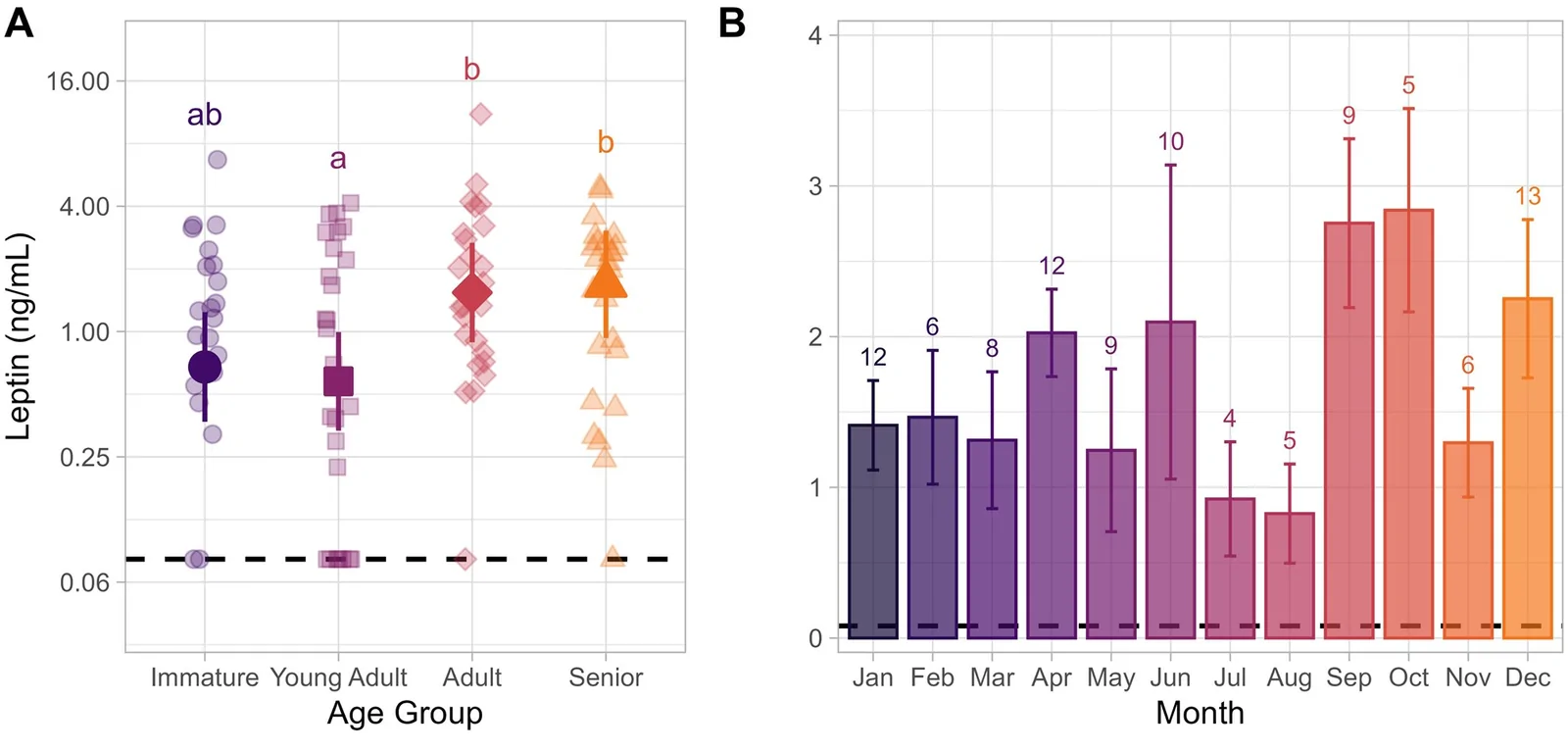
Leptin concentrations (ng/mL) across age groups in female polar bears (*Ursus maritimus*). Each error plot depicts individual raw data points for four age groups: Immature (circles), Young Adult (squares), Adult (diamonds), and Senior (triangles). Large points with error bars represent the model-adjusted means ± 95% confidence limits. Different lowercase letters above error plots indicate statistically significant differences among groups (*P *< 0.05). Groups sharing the same letter are not significantly different (Young Adult vs. Adult, *P* = 0.007; Young Adult vs. Senior, *P *= 0.009). (B) Monthly variation in Leptin concentrations (ng/mL) in female polar bears (*Ursus maritimus*). Each bar represents mean monthly Leptin levels from January to December. Error bars represent ± standard error of measurement (SEM). Sample sizes for each month are indicated above the corresponding error bars. The dashed, horizontal line represents the minimum detectable limit (MDL; 0.0806 ng/mL). No statistical analyses were performed for monthly comparisons.

Leptin concentrations exceeded the MDL of 0.0806 ng/mL in all but 11 samples, ranging 0.22 to 11.1 ng/mL. Four additional samples lacked leptin values due to insufficient serum for reanalysis after repeat runs were needed to resolve CV issues. Overall mean leptin concentration was 1.78 ± 0.17 ng/mL. Leptin concentrations varied among age groups (overall model *P *= 0.001; Figure 2A), with the greatest values observed in senior (model-adjusted mean: 1.69 ± 0.50 ng/mL) and adult females (1.54 ± 0.42 ng/mL), compared to young adults (0.58 ± 0.16 ng/mL) and immature individuals (0.68 ± 0.20 ng/mL). Monthly patterns showed scattered peaks in early summer, with no consistent seasonality (Figure 2B). Neither season (model-adjusted means: winter: 1.02 ± 0.28 ng/mL; spring: 0.79 ± 0.20, summer: 0.98 ± 0.28; fall: 1.28 ± 0.35; overall model *P *= 0.43) nor parity (model-adjusted means: proven: 1.05 ± 0.32 ng/mL, not proven: 1.05 ± 0.34; overall model *P *= 0.88) were significant predictors in mixed-effects models.

### Luteinizing hormone (LH)

**Figure 3:**
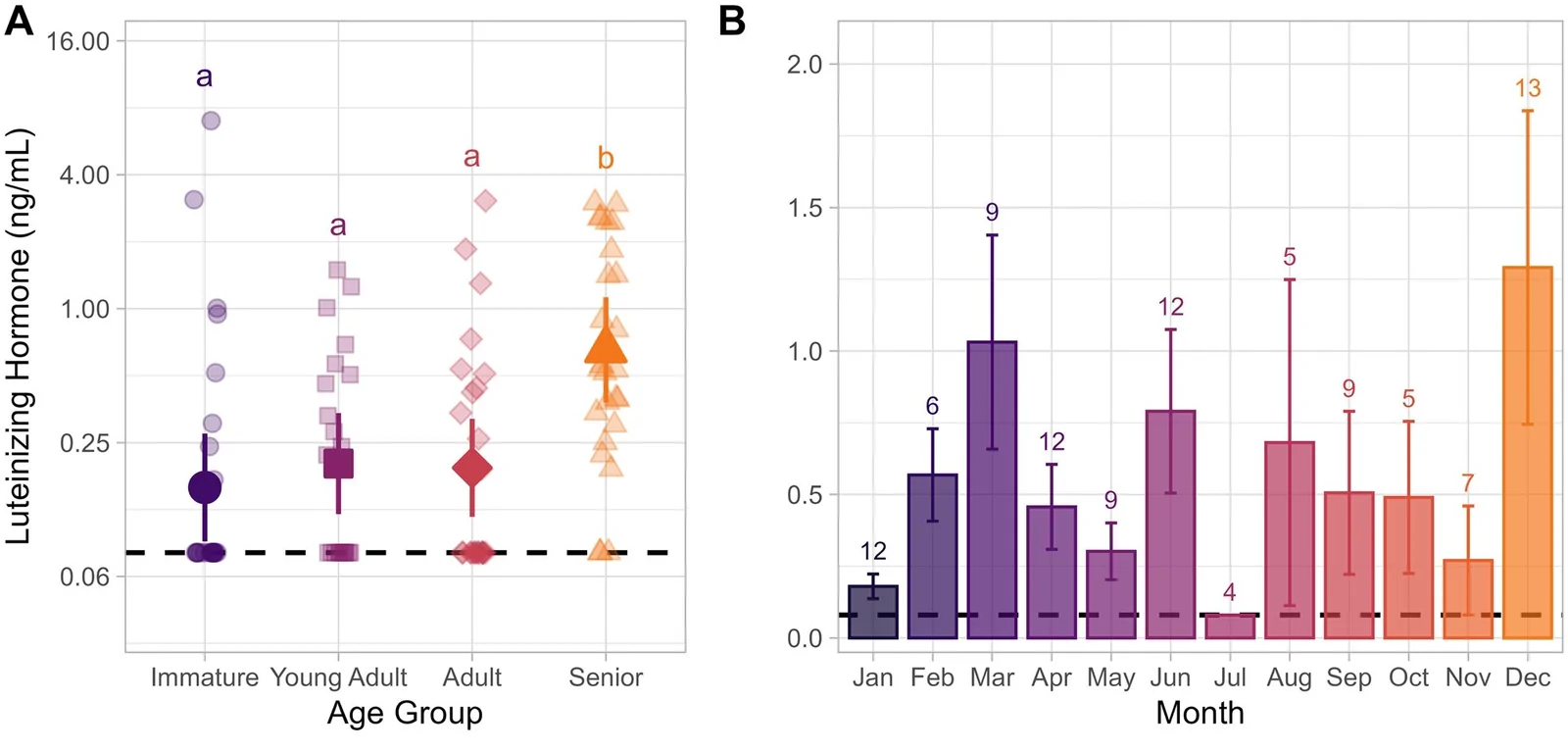
(A) Variation in Luteinizing Hormone (LH) concentrations (ng/mL) across age groups in female polar bears (*Ursus maritimus*). Each error plot depicts individual raw data points for four age groups: Immature (circles), Young Adult (squares), Adult (diamonds), and Senior (triangles). Large points with error bars represent the model-adjusted means ± 95% confidence limits. Different lowercase letters above error plots indicate statistically significant differences among groups (*P *< 0.05). Groups sharing the same letter are not significantly different (Senior vs. Immature, *P *= 0.003; Senior vs. Young Adult, *P *= 0.005; Senior vs. Adult, *P *= 0.004). (B) Monthly variation in LH concentrations (ng/mL) in female polar bears. Each bar represents mean monthly LH levels from January to December. Error bars represent ± standard error of measurement (SEM). Sample sizes for each month are indicated above the corresponding error bars. The dashed, horizontal line represents the minimum detectable limit (MDL; 0.08 ng/mL). No statistical analyses were performed for monthly comparisons.

LH values were above the MDL (0.08 ng/mL) in 58 of 103 samples, these values ranged from 0.17 to 7.0 ng/mL. Average concentration was 0.60 ± 0.10 ng/mL. LH concentrations differed across age groups (overall model: *P *= 0.0002), with the greatest values observed in seniors (model-adjusted mean: 0.65 ± 0.18 ng/mL) while immature bears exhibited the lowest (0.16 ± 0.04 ng/mL). Monthly data revealed a peak in December (1.29 ± 0.55 ng/mL), and a smaller elevation in March (1.12 ± 0.37 ng/mL; Figure 3B). Neither season (model-adjusted means: winter: 0.25 ± 0.07 ng/mL, spring: 0.23 ± 0.06, summer: 0.19 ± 0.05, fall: 0.35 ± 0.09; overall model *P *= 0.42) nor parity (model-adjusted means: proven: 0.33 ± 0.09, not proven: 0.29 ± 0.08; overall model *P *= 0.92) were significant predictors in the full model.

### Prolactin (PRL)

**Figure 4:**
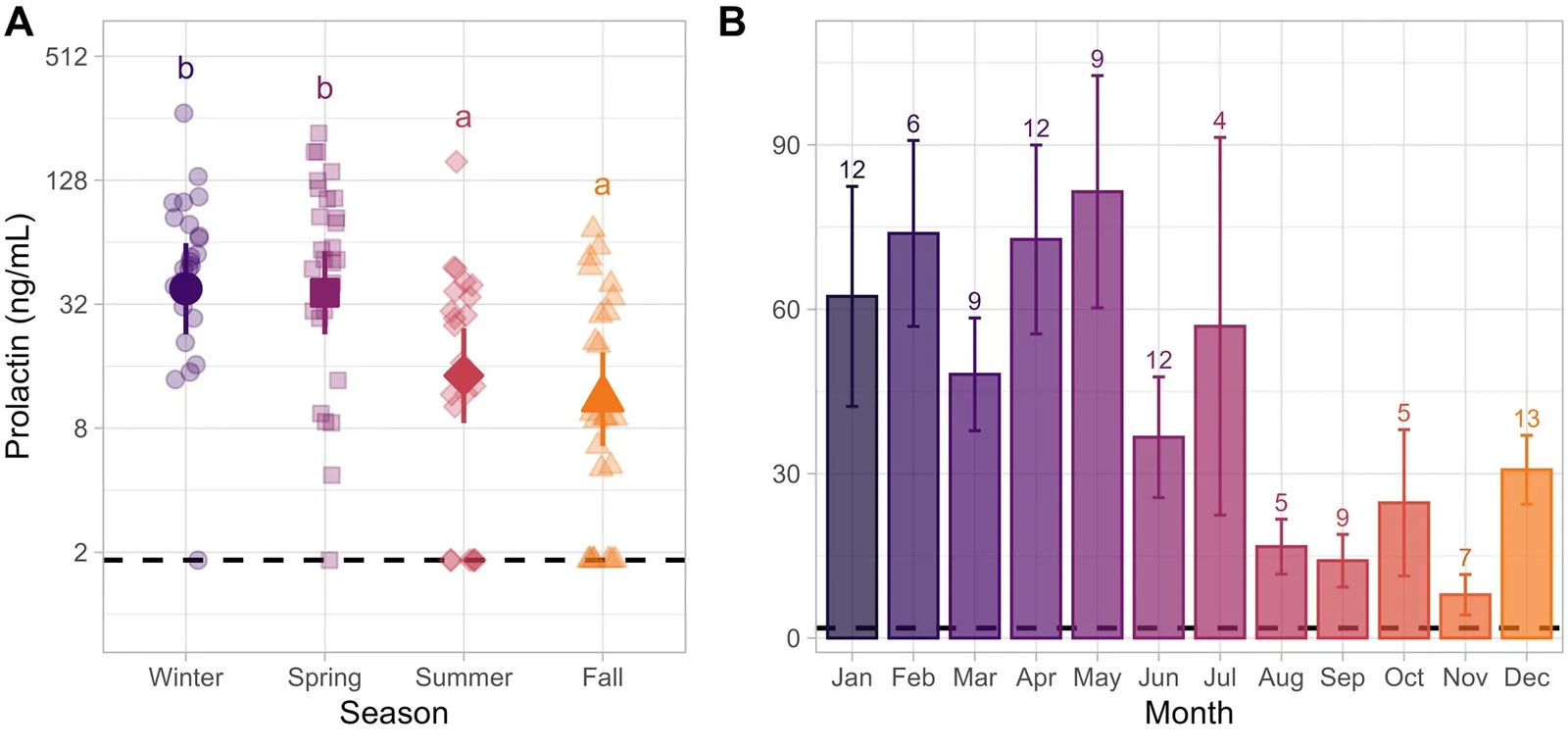
(A) Seasonal variation in prolactin concentrations (ng/mL) in female polar bears (*Ursus maritimus*). Individual data points represent raw measurements across four seasons: Winter (circles), Spring (squares), Summer (diamonds), and Fall (triangles). The solid point in the middle of each error plot represents the model-adjusted means calculated from the corresponding linear mixed-effects model; the error bars represent the upper and lower 95% confidence limit. Different lowercase letters above error plots indicate statistically significant differences among groups (*P *< 0.05). Groups sharing the same letter are not significantly different (Winter vs. Summer, *P *= 0.03; Winter vs. Fall, *P *= 0.006; Spring vs. Summer, *P *= 0.03; Spring vs. Fall, *P *= 0.003). (B) Monthly variation in prolactin concentrations (ng/mL) in female polar bears. Each bar represents mean monthly prolactin levels from January to December. Error bars represent ± standard error of measurement (SEM). Sample sizes for each month are indicated above the corresponding error bars. The dashed, horizontal line represents the minimum detectable limit (MDL; 1.83 ng/mL). No statistical analyses were performed for monthly comparisons.

Prolactin concentrations were below the MDL (1.83 ng/mL) in 15 samples; all others fell between 4.73 and 270.9 ng/mL. The overall mean was 45.49 ± 4.80 ng/mL. PRL concentrations varied by season (overall model *P *= 0.0001), with the greatest values observed in winter and spring (model-adjusted means: 38.05 ± 9.71 and 36.38 ± 8.49 ng/mL, respectively), compared to summer and fall (14.41 ± 3.84 and 11.08 ± 2.92 ng/mL, respectively; Figure 4A). Monthly data showed elevation spanning from January to July, aligning with polar bear breeding season (Figure 4B). Age group was a significant predictor of prolactin concentrations (overall model *P *= 0.04), but post hoc pairwise comparisons revealed no significant differences between groups (model-adjusted means: Immature: 17.15 ± 4.69 ng/mL, young adult: 36.54 ± 9.45, adult: 23.40 ± 5.81, senior: 15.07 ± 3.92; all pairwise comparisons *P *> 0.06). Prolactin concentrations were not impacted by parity (model-adjusted means: proven: 27.49 ± 6.39 ng/mL, not proven: 19.17 ± 5.07; overall model *P *= 0.58).

## Discussion

To our knowledge, this is the first study to characterize circulating FSH, GH, leptin, LH, and PRL in female polar bears, and to apply multiplex immunoassay technology to evaluate reproductive hormones in this species. This approach establishes a method for hormone quantification using minimal serum volumes, which is particularly valuable for monitoring this threatened species.

### Follicle stimulating hormone and luteinizing hormone

Follicle stimulating hormone (FSH) and luteinizing hormone (LH) are key components of the hypothalamic-pituitary-gonadal (HPG) axis, regulating ovarian function through coordinated, pulsatile secretion. FSH promotes follicular growth and recruitment, while LH stimulates ovulation and luteal function, with both hormones modulated by steroidal feedback and environmental cues in seasonally breeding species (Beltran-Frutos et al. 2022). In this study, most FSH values fell below the assay’s detection threshold, limiting interpretability. Although validation criteria (e.g. parallelism and recovery) were met, the assay lacked sufficient sensitivity to reliably quantify FSH in polar bears. Limited detectability likely reflects both analytical and biological constraints, such as the inability to independently optimize analytes within a multiplex platform, low circulating concentrations, and/or reduced antibody cross-reactivity. All detectable values occurred in winter and early spring, with greatest concentrations in December and January, consistent with the onset of follicular development during the pre-breeding period, observed in other species, including canids (Concannon et al. 2009). However, interpretation is further constrained by the pulsatile nature of FSH secretion and opportunistic sampling, which may not capture transient peaks, as well as the lack of ovarian activity confirmation. Targeted, single-analyte assays may therefore be required to improve sensitivity and fully characterize FSH dynamics in polar bears.

LH concentrations were more reliably detected and exhibited a significant age-related increase, with the greatest values observed in senior bears. This mirrors endocrine aging patterns in humans, where estrogen decline reduces negative feedback on the HPG axis and triggers compensatory LH elevation (Lenton et al. 1988; Ahmed Ebbiary et al. 1994). Although estradiol was not measured here, earlier studies have reported age-related declines in AMH (Tompros et al. 2025) and signs of reproductive senescence after age 20 years (Naciri et al. 2022), suggesting that elevated LH may reflect waning ovarian responsiveness in older polar bears. However, approximately 44% of LH concentrations fell below the MDL and required value substitution, which may reduce within-group variance and influence the magnitude of observed differences. As such, the strength of this age-related pattern should be interpreted with appropriate caution. One outlier LH value from a 1-month-old cub also warrants mention, as it may represent a transient postnatal hormone surge associated with early activation of the HPG axis, a pattern observed in other mammals including humans (Lanciotti et al. 2018; Devillers et al. 2022), though additional sampling across this age class is required to determine whether this pattern is consistent.

Season and reproductive history did not significantly predict LH concentrations; however, mean values were greatest in December, with a smaller elevation observed in March. While month was not included in the statistical models, this pattern may reflect increased pituitary output preceding ovulation, consistent with seasonal activation of the reproductive axis in other species (Beltran-Frutos et al. 2022). This timing also aligns with the seasonal rise in GH and PRL observed in this study, suggesting broader endocrine activation during the winter months in preparation for reproduction. A seasonal increase in LH has also been reported in male polar bears, coinciding with springtime elevations in testosterone (Howell-Skalla et al. 2002), supporting the potential for photoperiodic regulation of the HPG axis in this species. However, interpretation of mean LH concentrations in females is complicated by the pulsatile nature of LH secretion, where brief, high-amplitude surges may be missed in samples collected opportunistically rather than through intensive, time-series sampling. The absence of clear seasonal patterns in females may therefore reflect limitations in sampling frequency or power rather than a true lack of effect. Previous work in American black bears (*Ursus americanus*) has likewise failed to consistently identify seasonal variation in LH (Tsubota et al. 1998; Howell-Skalla et al. 2000), though differences in study design complicate comparisons. LH and FSH remain promising targets for monitoring reproductive transitions in polar bears; however, improved assay sensitivity and more targeted sampling approaches will be necessary to fully resolve their dynamics.

### Growth hormone

Serum growth hormone (GH) concentrations in females followed a clear seasonal trajectory, with peak levels observed in winter, followed by a decline through spring and summer, and a modest rise beginning in late fall. In the wild, pregnant polar bears den during the fall and winter months, fasting while gestating and lactating, and relying almost entirely on stored lipids to meet their energetic demands (Atkinson et al. 1996). Because the individuals in this study were not pregnant and experienced minimal to moderate, voluntary reductions in food intake relative to wild denning females, the observed winter GH peak may reflect combined regulation by photoperiod and metabolic state rather than a single factor alone. In another seasonally reproductive carnivore, the arctic fox, GH secretion responds to both cues, particularly during prolonged fasting or torpor when GH helps mobilize fat reserves and preserve lean tissue to maintain energy balance (Fuglei et al. 2004). Although polar bears do not hibernate in the traditional sense, some individuals displayed denning behavior and voluntarily reduced food intake in late fall, paralleling wild seasonal patterns and potentially engaging similar GH-mediated pathways. However, not all bears showed these behaviors, suggesting that photoperiodic rhythms may sustain GH elevation even when metabolic demands are reduced. Together, these findings support a dual regulatory role for GH influenced by both photoperiod and metabolic state.

Comparable GH patterns have been reported in other carnivores with seasonal metabolic adaptations. Captive raccoon dogs (*Nyctereutes procyonoides*), for instance, exhibited elevated GH and leptin concentrations in winter even when fed ad libitum, suggesting strong seasonal regulation (Nieminen et al. 2002). In contrast, GH elevations in wild raccoon dogs were less consistent and appeared to be modulated by body condition (Asikainen et al. 2004), indicating that metabolic status can influence the expression of otherwise photoperiod-driven rhythms. Among hibernating bears, seasonal regulation of the GH/Insulin-like Growth Factor-I (IGF-I) axis, a pathway in which GH stimulates IGF-I production to promote tissue growth, protein synthesis, and metabolic homeostasis, is well documented. In black bears, IGF-I concentrations declined early in hibernation and rebounded in late winter, likely due to changes in GH sensitivity or binding proteins (Blumenthal et al. 2011). Scandinavian brown bears (*Ursus arctos*) exhibit similar seasonal patterns in anabolic and catabolic balance mediated through GH and IGF-I (Fröbert et al. 2022). These examples suggest that seasonal modulation of GH signaling may represent a shared physiological feature among carnivores with pronounced seasonal metabolic shifts. Further investigation into how GH integrates with other endocrine pathways, such as melatonin, PRL, and thyroid hormones, which are known to mediate seasonal physiology across a range of mammalian species (Goldman 2001) could enhance our understanding of polar bear metabolic regulation under both wild and managed conditions.

### Leptin

Leptin is a metabolic hormone predominantly produced by white adipose tissue, serving as a key regulator of energy balance (Ahima and Flier 2000) and a permissive signal for reproductive function (Barash et al. 1996; Childs et al. 2021). In this study, serum leptin concentrations increased with age, with the greatest levels observed in senior bears. Neither reproductive history nor season significantly predicted leptin values. In wild polar bears, older females tend to enter dens with greater fat stores than younger ones, possibly due to enhanced foraging efficiency or the selective survival of individuals better able to accumulate energy reserves (Atkinson and Ramsay 1995). While body mass data were not available, greater levels in older individuals may reflect these cumulative energy reserves. Leptin is also an appetite suppressant, and its greater concentrations in older bears may reflect life-history strategies: senior females, which are less likely to reproduce, may not require the same hyperphagic drive as younger individuals. This pattern highlights how leptin may contribute to age-related energy allocation strategies in polar bears. However, the absence of comparable wild leptin datasets limits evaluation of whether these patterns reflect species-wide trends or conditions specific to managed populations.

Leptin–adiposity relationships have been well characterized in carnivores, though interspecific variability suggests species- and context-dependent regulation. Positive correlations between leptin and fat mass have been documented in several species, including European brown bears (Hissa et al. 1998), American black bears (Spady et al. 2009), as well as other members of the caniformia suborder including blue foxes (Mustonen et al. 2005) and dogs (Sagawa et al. 2002). However, some seasonally reproductive species, such as raccoon dogs (Nieminen et al. 2001) and northern elephant seals (Ortiz et al. 2001), have reported no clear relationship between leptin and body mass or fat stores. Such variability may reflect species-specific differences in leptin sensitivity or how tightly leptin is linked to metabolic state under different environmental conditions. In polar bears under managed care, seasonal variation in leptin may be minimized relative to wild populations due to consistent food availability and moderated seasonal weight loss, illustrating how species- and context-dependent factors can influence leptin regulation.

Leptin’s reproductive role is often associated with signaling energy sufficiency, thereby facilitating puberty, and sustaining fertility in adults, although this relationship varies across species and physiological contexts (Barash et al. 1996; Evans et al. 2022). In some seasonal breeders, such as ewes and Siberian hamsters, reproductive suppression occurs in response to photoperiod regardless of leptin levels, likely due to seasonal changes in leptin sensitivity or hypothalamic signaling (Atcha et al. 2000; Adam and Mercer 2001; Miller et al. 2002). A similar mechanism may apply here: despite seasonal declines in body mass, leptin concentrations did not change across seasons, suggesting that photoperiod-driven reproductive or metabolic processes may proceed via shifts in receptor expression or tissue responsiveness rather than changes in circulating leptin itself (Klingenspor et al. 2000; Adam and Mercer 2001). Consistent with this interpretation, the lack of seasonal leptin variation contrasts with the clear winter elevation of GH observed herein. These divergent seasonal profiles of GH and leptin suggest that GH may be more directly entrained to photoperiod or circannual rhythms, whereas leptin’s role in seasonal physiology may depend on changes in sensitivity rather than absolute concentration. Although photoperiod varies with latitude, the relatively narrow geographic range represented in this study limited our ability to evaluate such effects independently; additionally, given the distinct seasonal physiology of polar bears, these relationships remain unclear. Further investigation into GH–leptin interactions may clarify how these hormones coordinate energy management and reproductive readiness in polar bears.

### Prolactin

Prolactin (PRL) concentrations in female polar bears exhibited a clear seasonal pattern, with elevated levels during winter and spring followed by a decline through summer and fall. The transition aligns with the species’ reproductive timeline: mating occurs in spring, followed by embryonic diapause and presumed implantation in the fall. The observed decline in PRL parallels this shift and supports the hypothesis proposed in black bears that high PRL concentrations may maintain diapause, while a reduction in PRL acts as a permissive signal for implantation (Tsubota et al. 1998; Sato et al. 2001). Similar patterns occur in the tammar wallaby (*Macropus eugenii*), a well-studied marsupial with seasonal embryonic diapause, where PRL secretion is entrained by photoperiod and elevated levels suppress luteal activity to maintain embryonic quiescence (Tyndale-Biscoe and Hinds 1984; Hinds and Tyndale-Biscoe 2013). In contrast, some mammals with delayed implantation, including mink (Murphy et al. 1981; Douglas et al. 1998) and western spotted skunks (*Spilogale gracilis*; Berria et al. 1989), also exhibit photoperiodic regulation of reproduction, but PRL functions differently: it stimulates the corpus luteum to resume progesterone production and initiate implantation. Thus, while seasonal cues such as photoperiod synchronize reproductive timing across taxa, PRL’s downstream role diverges, either delaying implantation by suppressing luteal activity, or promoting implantation through luteal activation. This functional variability highlights PRL’s central yet context-dependent role in coordinating reproductive timing with environmental seasonality.

PRL concentrations varied significantly across age groups at the overall model level, with the greatest values observed in young adult females followed by a gradual decline across older age groups. However, pairwise comparisons were not statistically significant, although the contrast between immature and senior individuals approached significance (*P *= 0.055). Similar age-related declines in PRL have been described in women in parallel with decreasing estrogen concentrations (Vekemans and Robyn 1975; Randolph et al. 2004), suggesting a regulatory link between PRL and ovarian steroids. While a comparable decline in estrogens has not been confirmed in polar bears, evidence of reproductive senescence, particularly after age 20, has been reported (Naciri et al. 2022). Supporting this, AMH, a marker of ovarian reserve, declined with age in this species (Tompros et al. 2025), indicating a progressive loss of follicular function. The convergence of age-related declines in AMH and PRL and the observed increase in LH supports the hypothesis that multiple endocrine pathways are altered during reproductive aging in female polar bears. These findings further highlight the utility of multiplexed hormone profiling for detecting subtle but coordinated shifts in endocrine function and suggest that no one hormone should be used as a stand-alone indicator of reproductive potential.

Additionally, reproductive experience may influence PRL levels. In birds, such as the wandering albatross (*Diomedea exulans*), PRL concentrations are lower in first-time breeding females than in experienced individuals, with an even more pronounced experience-related increase observed in males (Angelier et al. 2006). While these findings provide a speculative point of comparison for experience-related endocrine modulation, comparable evidence in mammals is limited. In many mammalian species, age and reproductive experience are often confounded, making it difficult to separate their effects. Although the present dataset distinguished proven from unproven breeders, parity information was insufficient to evaluate finer-scale experience effects or disentangle experience from age-related influences. Age- and experience-related modulation of PRL secretion remains a promising avenue for future work, with potential implications for understanding reproductive senescence and lifetime reproductive output in this species.

### Limitations

This study’s retrospective and opportunistic design introduce several limitations that should be considered when interpreting the findings. Samples were collected from zoo-housed polar bears with consistent access to food and stable housing conditions, which likely buffered against the seasonal fluctuations in energy balance and associated hormone dynamics that may occur in the wild. Body condition data were not available, limiting the ability to evaluate energetic influences on hormone concentrations. Although parity was recorded, females did not produce cubs during the year of sampling, and none were confirmed pregnant, so profiles do not reflect endocrine changes associated with gestation or peri-implantation physiology. Early pregnancy loss cannot be ruled out but was not documented. In addition, records did not consistently indicate whether bears were housed with conspecifics, which may influence reproductive hormone activity. Variation in sample numbers per individual further constrained interpretability.

Assay sensitivity, particularly for FSH, limited detection of physiological concentrations and may have reduced the ability to detect subtle variation. Although FSH met analytical validation criteria, the assay lacked sufficient sensitivity for routine quantification in this species. Differences in assay performance among analytes may reflect structural characteristics of the hormones. The poorest detectability was observed for glycoprotein hormones (FSH and LH, as well as the excluded TSH), which are heterodimeric and subject to variable glycosylation, producing multiple circulating isoforms that can affect antibody binding. In contrast, analytes with stronger performance (GH, leptin, and PRL) are single-chain proteins. These structural differences, combined with the use of antibodies raised against canid and rat targets, may reduce cross-reactivity and contribute to limited sensitivity. Glycoprotein hormones may therefore require additional scrutiny during assay validation.

Despite these constraints, the study revealed consistent age- and season-related effects across several analytes. Future research would benefit from prospective, longitudinal sampling of both captive and wild females, paired with comprehensive records of physiological status, social context, and confirmed reproductive outcomes. Specifically, comparisons between pregnant and non-pregnant females during key reproductive windows, such as implantation, will be essential to refine the interpretation and application of these hormonal biomarkers. However, such efforts remain logistically difficult, as sampling from wild polar bears, particularly during denning, is limited and rarely feasible. Methodological improvements should focus on increasing sensitivity for low-abundance analytes (e.g. FSH and LH), including targeted single-analyte assays, optimization of antibody specificity, and evaluation of alternative platforms. Controlled studies examining cross-reactivity, matrix effects, and species-specific hormone structure would further clarify the drivers of limited detectability, particularly for glycoprotein hormones. Although such efforts will likely require species-specific reagents, including purified polar bear hormone standards.

## Conclusions

As polar bears face increasing environmental pressures and reproductive challenges, the development of sensitive, minimally invasive tools for assessing reproductive function is crucial for conservation efforts. This study validated the use of multiplex immunoassays capable of simultaneously measuring multiple hormones from small serum volumes, offering a valuable framework for monitoring endocrine status in this threatened species. Hormone patterns for many analytes varied by age and season, aligning with expectations for a seasonal breeder and highlighting the complexity of polar bear reproductive endocrinology. Continued refinement and validation of these biomarkers will be key to improving our ability to monitor and support polar bear reproductive success in a rapidly changing environment.

## Supplementary Material

skag273_Supplementary_Data

## Data Availability

The data underlying this article are available in the article and in its online supplementary material.

## Abbreviations

ACTH: adrenocorticotropic hormone
AMH: anti-Müllerian hormone
FSH: follicle stimulating hormone
GH: growth hormone
IGF-I: insulin-like growth factor-I
LH: luteinizing hormone
PRL: prolactin
TSH: thyroid stimulating hormone
