# Supplementary material for: Characterization of serum follicle stimulating hormone, growth hormone, leptin, luteinizing hormone, and prolactin in female polar bears (Ursus maritimus) via multiplex immunoassay
Source: J Anim Sci. 2026 Aug 31;104:skag273. doi: 10.1093/jas/skag273 (PMC13570595; doi:10.1093/jas/skag273)
Supplement: Supplementary file 1 [file skag273_supplementary_data.zip › Supplementary B.docx]

Supplementary B:

Figure S1. (A) Seasonal variation in follicle stimulating hormone (FSH) concentrations (ng/mL) in female polar bears (*Ursus maritimus*). Individual data points represent raw measurements across four seasons: Spring (purple circles), Summer (maroon squares), Fall (red diamonds), and Winter (orange triangles). The solid point in the middle of each error plot represents the model-adjusted means calculated from the corresponding linear mixed-effects model; the error bars represent the upper and lower 95% confidence limit. (B) Monthly variation in FSH concentrations (ng/mL) in female polar bears. Each bar represents mean monthly FSH levels from January to December. Error bars represent ± standard error of measurement (SEM). Sample sizes for each month are indicated above the corresponding error bars. The dashed, horizontal line represents the minimum detectable limit (MDL; 2.3 0.95 ng/mL). No statistical analyses were performed.

|  | FSH (ng/mL) | GH (ng/mL) | Leptin (ng/mL) | LH (ng/mL) | Prolactin (ng/mL) |
| --- | --- | --- | --- | --- | --- |
| 1 freeze/thaw | 5.3 | 5.2 | 2.56 | 0.27 | 25.8 |
| 2 freeze/thaw | 6.3 | 5.2 | 2.95 | 0.26 | 30.7 |
| 3 freeze/thaw | 4.8 | 5.6 | 2.77 | 0.28 | 32.7 |
| 4 freeze/thaw | 6.1 | 5.9 | 2.84 | 0.30 | 27.4 |

Figure S2. Freeze–thaw stability of multiplex immunoassay analytes in female polar bear (*Ursus maritimus*) serum across repeated freeze–thaw cycles. Concentrations of follicle-stimulating hormone (FSH), growth hormone (GH), leptin, luteinizing hormone (LH), and prolactin were measured following repeated freeze–thaw cycles (1–4 cycles) to assess assay stability.
