# Supplementary material for: Characterization of serum follicle stimulating hormone, growth hormone, leptin, luteinizing hormone, and prolactin in female polar bears (Ursus maritimus) via multiplex immunoassay
Source: J Anim Sci. 2026 Aug 31;104:skag273. doi: 10.1093/jas/skag273 (PMC13570595; doi:10.1093/jas/skag273)
Supplement: Supplementary file 1 [file skag273_supplementary_data.zip › Supplementary A.docx]

**Supplementary A:** Individual- and sample-level metadata and serum hormone concentrations (ng/mL) in female polar bears (*Ursus maritimus*). FSH = follicle stimulating hormone, GH = growth hormone, LH = luteinizing hormone, PRL = prolactin.

| **Bear** | **Birth Origin** | **Age Class** | **Age (y)** | **Month** | **Season** | **Proven Parity** | **Breeding Season** | **FSH** | **GH** | **Leptin** | **LH** | **PRL** |
| --- | --- | --- | --- | --- | --- | --- | --- | --- | --- | --- | --- | --- |
| A | zoo | Immature | 2.24 | February | Winter | juvenile | breeding | 11.30 | 0.25 | 3.13 | 0.08 | 27.37 |
| A | zoo | Immature | 1.06 | November | Fall | juvenile | non-breeding | 0.95 | 0.13 | 0.64 | 0.08 | 1.83 |
| B | zoo | Adult | 18.82 | October | Fall | proven | non-breeding | 0.95 | 0.01 | 2.76 | 0.08 | 5.23 |
| B | zoo | Adult | 14.19 | February | Winter | proven | breeding | 0.95 | 15.25 | 0.62 | 0.34 | 31.10 |
| B | zoo | Adult | 18.61 | July | Summer | proven | non-breeding | 0.95 | 0.01 | 0.52 | 0.08 | 1.83 |
| B | zoo | Adult | 18.74 | September | Summer | proven | non-breeding | 0.95 | 0.01 | 1.33 | 0.08 | 1.83 |
| C | zoo | Senior | 21.49 | May | Spring | unproven | breeding | 0.95 | 0.38 | 4.76 | 0.39 | 29.80 |
| C | zoo | Senior | 22.14 | January | Winter | unproven | breeding | 0.95 | 2.91 | 2.87 | 0.08 | 16.26 |
| C | zoo | Young Adult | 11.68 | July | Summer | unproven | non-breeding | 0.95 | 0.39 | 0.44 | 0.08 | 27.32 |
| C | zoo | Adult | 13.79 | August | Summer | unproven | non-breeding | 0.95 | 1.29 | 2.02 | 0.08 | 10.20 |
| C | zoo | Adult | 16.05 | December | Fall | unproven | non-breeding | 0.95 | 0.65 | 4.10 | 0.08 | 34.42 |
| D | zoo | Young Adult | 8.33 | March | Spring | unproven | breeding | 0.95 | 0.22 | 0.08 | 0.08 | 60.24 |
| D | zoo | Young Adult | 11.11 | January | Winter | proven | breeding | 0.95 | 0.23 | 0.08 | 0.08 | 100.20 |
| D | zoo | Senior | 26.24 | February | Winter | proven | breeding | 0.95 | 27.66 | 1.97 | 0.66 | 77.97 |
| E | zoo | Immature | 4.06 | December | Fall | juvenile | non-breeding | 0.95 | 7.63 | 1.30 | 0.08 | 48.38 |
| E | zoo | Immature | 1.96 | November | Fall | juvenile | non-breeding | 0.95 | 0.13 | 1.16 | 0.08 | 29.18 |
| E | zoo | Immature | 1.56 | June | Spring | juvenile | non-breeding | 0.95 | 0.27 | 0.46 | 0.08 | 27.33 |
| F | zoo | Adult | 18.23 | January | Winter | unproven | breeding | 0.95 | 2.02 | 0.79 | 0.08 | 51.57 |
| F | zoo | Young Adult | 6.57 | June | Spring | unproven | non-breeding | 0.95 | 0.81 | 0.08 | 0.22 | 140.90 |
| F | zoo | Young Adult | 10.35 | March | Winter | unproven | breeding | 0.95 | 1.05 | 0.08 | 0.28 | 39.16 |
| G | zoo | Senior | 22.33 | March | Winter | proven | breeding | 0.95 | 10.82 | 0.29 | 2.45 | 49.08 |
| G | zoo | Senior | 20.19 | January | Winter | proven | breeding | 0.95 | 11.47 | 1.58 | 0.38 | 270.95 |
| G | zoo | Senior | 28.35 | March | Winter | proven | breeding | 0.95 | 54.92 | 2.14 | 1.84 | 84.17 |
| G | zoo | Adult | 15.10 | December | Fall | proven | non-breeding | 0.95 | 0.91 | 0.97 | 1.30 | 73.84 |
| G | zoo | Young Adult | 8.17 | January | Winter | proven | breeding | 0.95 | 12.67 | 1.03 | 0.08 | 20.87 |
| G | zoo | Young Adult | 8.24 | January | Winter | proven | breeding | 0.95 | 2.76 | 0.08 | 0.33 | 55.99 |
| G | zoo | Young Adult | 9.27 | February | Winter | proven | breeding | 0.95 | 7.97 | 0.08 | 0.51 | 133.21 |
| G | zoo | Senior | 22.33 | March | Winter | proven | breeding | 0.95 | 8.78 | --- | 2.99 | 47.56 |
| H | zoo | Adult | 19.11 | January | Winter | unproven | breeding | 0.95 | 0.77 | 1.32 | 0.51 | 36.82 |
| H | zoo | Senior | 23.08 | December | Winter | unproven | non-breeding | 21.90 | 8.91 | 0.81 | 0.40 | 68.49 |
| I | zoo | Adult | 17.61 | August | Summer | unproven | non-breeding | 0.95 | 0.01 | 0.90 | 0.08 | 16.62 |
| I | zoo | Adult | 17.64 | August | Summer | unproven | non-breeding | 0.95 | 0.01 | 0.08 | 0.08 | 1.83 |
| I | zoo | Immature | 1.50 | June | Summer | juvenile | non-breeding | 0.95 | 2.83 | --- | 0.95 | 48.38 |
| I | zoo | Immature | 0.22 | March | Winter | juvenile | breeding | 0.95 | 17.57 | 0.08 | 0.31 | 1.83 |
| I | zoo | Immature | 5.40 | May | Spring | juvenile | breeding | 0.95 | 2.18 | 0.08 | 0.69 | 29.66 |
| J | zoo | Senior | 28.56 | June | Summer | unproven | non-breeding | 0.95 | 0.01 | 2.89 | 0.19 | 1.83 |
| J | zoo | Senior | 29.55 | June | Summer | unproven | non-breeding | 0.95 | 0.01 | 2.36 | 0.81 | 1.83 |
| J | zoo | Senior | 29.05 | December | Winter | unproven | non-breeding | 0.95 | 2.56 | 2.52 | 0.22 | 35.52 |
| K | zoo | Immature | 0.07 | December | Fall | juvenile | non-breeding | 0.95 | 13.44 | 0.32 | 7.00 | 1.83 |
| K | zoo | Immature | 0.50 | May | Spring | juvenile | non-breeding | 0.95 | 5.05 | 0.77 | 0.08 | 104.60 |
| K | zoo | Young Adult | 8.44 | April | Spring | unproven | breeding | 7.80 | 28.79 | 1.13 | 0.08 | 58.68 |
| K | zoo | Young Adult | 11.13 | January | Winter | unproven | breeding | 9.60 | 13.08 | 2.21 | 0.08 | 14.99 |
| K | zoo | Adult | 12.14 | January | Winter | unproven | breeding | 0.95 | 51.54 | 1.31 | 0.08 | 54.21 |
| K | zoo | Adult | 13.16 | January | Winter | unproven | breeding | 0.95 | 29.84 | 2.06 | 0.08 | 47.56 |
| K | zoo | Adult | 14.15 | January | Winter | unproven | breeding | 0.95 | 31.59 | 3.22 | 0.19 | 37.51 |
| K | zoo | Adult | 16.80 | September | Summer | unproven | non-breeding | 0.95 | 3.61 | 5.10 | 0.08 | 11.67 |
| L | zoo | Immature | 2.58 | June | Spring | juvenile | non-breeding | 0.95 | 0.01 | --- | 0.52 | 47.46 |
| L | zoo | Immature | 2.37 | March | Spring | juvenile | breeding | 0.95 | 0.24 | 2.46 | 0.08 | 37.64 |
| L | zoo | Immature | 2.87 | September | Fall | juvenile | non-breeding | 0.95 | 0.12 | 3.25 | 0.08 | 8.75 |
| M | zoo | Immature | 5.27 | February | Winter | juvenile | breeding | 0.95 | 9.83 | 1.15 | 0.57 | 106.33 |
| M | zoo | Immature | 3.92 | October | Fall | juvenile | non-breeding | 0.95 | 3.58 | 3.24 | 0.08 | 1.83 |
| M | zoo | Immature | 3.45 | April | Spring | juvenile | breeding | 0.95 | 6.20 | 1.26 | 0.24 | 83.81 |
| M | zoo | Immature | 0.62 | June | Spring | juvenile | non-breeding | 0.95 | 3.43 | 0.96 | 0.08 | 8.60 |
| N | zoo | Adult | 17.98 | November | Fall | proven | non-breeding | 0.95 | 1.22 | --- | 0.08 | 1.83 |
| N | zoo | Senior | 26.98 | November | Fall | proven | non-breeding | 0.95 | 6.73 | 2.67 | 1.41 | 1.83 |
| O | zoo | Young Adult | 6.40 | May | Spring | unproven | breeding | 0.95 | 2.41 | 0.30 | 0.08 | 8.50 |
| O | zoo | Young Adult | 6.65 | August | Summer | unproven | non-breeding | 0.95 | 2.07 | 0.69 | 0.22 | 29.58 |
| O | zoo | Young Adult | 11.83 | October | Fall | unproven | non-breeding | 0.95 | 2.33 | 3.66 | 1.50 | 53.82 |
| O | zoo | Immature | 4.96 | November | Fall | juvenile | non-breeding | 0.95 | 1.40 | 0.55 | 0.08 | 5.09 |
| O | zoo | Adult | 19.34 | April | Spring | unproven | breeding | 0.95 | 1.34 | 2.29 | 1.85 | 13.66 |
| P | zoo | Immature | 0.11 | December | Fall | juvenile | non-breeding | 0.95 | 7.18 | 0.08 | 3.09 | 8.98 |
| Q | zoo | Senior | 25.78 | September | Summer | proven | non-breeding | 0.95 | 2.48 | 4.93 | 2.46 | 11.81 |
| Q | zoo | Senior | 26.02 | December | Fall | proven | non-breeding | 0.95 | 2.51 | 3.56 | 2.57 | 20.87 |
| R | zoo | Young Adult | 9.39 | May | Spring | proven | breeding | 0.95 | 4.70 | 0.22 | 0.24 | 216.28 |
| R | zoo | Immature | 2.89 | November | Fall | juvenile | non-breeding | 0.95 | 2.22 | 0.68 | 0.08 | 9.01 |
| R | zoo | Young Adult | 11.31 | April | Spring | proven | breeding | 0.95 | 0.42 | 3.19 | 0.08 | 116.63 |
| R | zoo | Adult | 13.81 | October | Fall | proven | non-breeding | 0.95 | 0.43 | 4.22 | 0.26 | 60.69 |
| R | zoo | Adult | 19.94 | December | Fall | proven | non-breeding | 0.95 | 0.34 | 3.99 | 0.73 | 40.00 |
| R | zoo | Senior | 24.98 | December | Fall | proven | non-breeding | 0.95 | 1.30 | 1.77 | 0.56 | 9.45 |
| R | zoo | Young Adult | 11.31 | April | Spring | proven | breeding | 0.95 | 0.35 | 3.01 | 0.08 | 103.61 |
| S | wild | Senior | 26.67 | September | Summer | proven | non-breeding | 0.95 | 0.70 | 0.91 | 1.42 | 12.82 |
| S | wild | Senior | 34.38 | May | Spring | proven | breeding | 0.95 | 1.56 | 0.08 | 0.89 | 52.61 |
| S | wild | Senior | 33.42 | June | Spring | proven | non-breeding | 0.95 | 0.67 | 0.24 | 2.56 | 50.88 |
| S | wild | Senior | 33.63 | August | Summer | proven | non-breeding | 0.95 | 1.45 | 0.43 | 2.95 | 25.22 |
| S | wild | Senior | 35.28 | April | Spring | proven | breeding | 0.95 | 0.59 | 2.69 | 0.34 | 4.73 |
| T | zoo | Young Adult | 10.15 | January | Winter | unproven | breeding | 22.40 | 7.27 | 0.38 | 0.19 | 41.19 |
| T | zoo | Immature | 4.86 | September | Fall | juvenile | non-breeding | 0.95 | 2.03 | 1.74 | 0.08 | 1.83 |
| T | zoo | Immature | 2.08 | December | Fall | juvenile | non-breeding | 0.95 | 1.46 | 6.70 | 0.17 | 20.24 |
| U | zoo | Adult | 19.41 | April | Spring | proven | breeding | 0.95 | 2.61 | 0.51 | 0.08 | 37.72 |
| U | zoo | Senior | 21.58 | June | Summer | proven | non-breeding | 0.95 | 5.66 | 0.46 | 0.08 | 28.32 |
| U | zoo | Senior | 27.02 | December | Fall | proven | non-breeding | 0.95 | 0.01 | 2.23 | 0.52 | 8.98 |
| U | zoo | Senior | 27.37 | April | Spring | proven | breeding | 0.95 | 0.01 | 2.54 | 0.30 | 40.48 |
| U | zoo | Senior | 30.27 | March | Winter | proven | breeding | 0.95 | 0.33 | 2.37 | 0.25 | 13.80 |
| U | zoo | Senior | 31.86 | October | Fall | proven | non-breeding | 0.95 | 0.14 | 0.31 | 0.54 | 1.83 |
| U | zoo | Senior | 30.77 | September | Summer | proven | non-breeding | 0.95 | 0.18 | 0.85 | 0.08 | 1.83 |
| V | wild | Young Adult | 10.14 | February | Winter | unproven | breeding | 0.95 | 7.48 | 1.84 | 1.26 | 67.04 |
| V | wild | Adult | 14.28 | April | Spring | unproven | breeding | 0.95 | 22.56 | 1.18 | 0.44 | 175.51 |
| V | wild | Adult | 14.53 | July | Summer | unproven | non-breeding | 0.95 | 17.90 | 0.69 | 0.08 | 157.36 |
| V | wild | Adult | 19.46 | June | Spring | unproven | non-breeding | 0.95 | 0.01 | 0.72 | 0.42 | 1.83 |
| W | zoo | Immature | 3.10 | December | Fall | juvenile | non-breeding | 0.95 | 1.70 | 0.93 | 0.08 | 28.22 |
| W | zoo | Immature | 2.01 | November | Fall | juvenile | non-breeding | 0.95 | 4.81 | 2.09 | 0.08 | 6.55 |
| X | zoo | Adult | 14.58 | June | Summer | proven | non-breeding | 0.95 | 2.39 | 1.72 | 3.06 | 47.62 |
| X | zoo | Adult | 14.59 | June | Summer | proven | non-breeding | 0.95 | 3.73 | 11.08 | 0.54 | 34.75 |
| X | zoo | Immature | 1.40 | April | Spring | juvenile | breeding | 0.95 | 0.01 | 1.37 | 1.01 | 52.80 |
| X | zoo | Young Adult | 10.43 | May | Spring | proven | breeding | 0.95 | 1.44 | 0.39 | 0.08 | 79.37 |
| Y | zoo | Immature | 5.73 | September | Summer | juvenile | non-breeding | 0.95 | 3.71 | 2.51 | 0.20 | 39.60 |
| Y | zoo | Immature | 3.52 | July | Summer | juvenile | non-breeding | 0.95 | 1.07 | 2.05 | 0.08 | 41.07 |
| Y | zoo | Young Adult | 6.19 | March | Winter | unproven | breeding | 0.95 | 0.76 | 3.00 | 1.01 | 99.64 |
| Y | zoo | Young Adult | 6.73 | September | Summer | unproven | non-breeding | 0.95 | 1.29 | 4.15 | 0.08 | 36.89 |
| Y | zoo | Young Adult | 11.29 | April | Spring | proven | breeding | 0.95 | 1.68 | 3.71 | 0.46 | 175.83 |
| Y | zoo | Adult | 13.41 | May | Spring | proven | non-breeding | 0.95 | 0.41 | 2.95 | 0.08 | 84.96 |
| Y | zoo | Senior | 23.34 | April | Spring | proven | breeding | 0.95 | 6.04 | 1.43 | 0.53 | 9.43 |
| Y | zoo | Young Adult | 9.39 | May | Spring | proven | non-breeding | 0.95 | 4.55 | 1.67 | 0.19 | 127.21 |
